# Use of firsekibart for acute gouty arthritis in a maintenance hemodialysis patient with multiple high-risk comorbidities: a case report

**DOI:** 10.3389/fphar.2026.1928098

**Published:** 2026-10-15

**Authors:** Jiaqi Chen, Ruoyu Tong, Meng Shen, Weidong Zhang, Yuanhang Huang

**Affiliations:** 1 The First Affiliated Hospital (The First Clinical Medical School) of Guangdong Pharmaceutical University, Guangzhou, China; 2 Nephrology Department, General Hospital of Southern Theater Command, Guangzhou, China

**Keywords:** acute gouty arthritis, case report, firsekibart, hemodialysis, IL-1β, stage 5 chronic kidney disease

## Abstract

**Objective:**

To describe the clinical course of Firsekibart treatment for acute gouty arthritis in a 51-year-old patient receiving maintenance hemodialysis with multiple high-risk comorbidities, focusing on the temporal relationship among dialysis, drug administration and the timing of symptom changes. This report adds to the limited evidence on using Firsekibart, a novel fully human monoclonal antibody targeting IL-1β, in patients receiving maintenance hemodialysis.

**Methods:**

We retrospectively reviewed the patient’s underlying diseases, acute gout manifestations, timing of Firsekibart administration relative to hemodialysis, visual analogue scale (VAS) pain scores, body temperature, and inflammatory markers. The clinical course was interpreted in the context of available clinical evidence on Firsekibart, experience with other IL-1 pathway agents in patients with advanced chronic kidney disease and/or receiving dialysis, and monoclonal antibody pharmacokinetics.

**Results:**

Pain relief was documented 6–7 h after Firsekibart administration, before the first post-dose hemodialysis session; however, tramadol and topical flurbiprofen had been administered within the preceding hour and may have contributed to the observed improvement. No worsening or rebound of pain was observed on the day of dialysis or the following day. No immediate hypersensitivity reaction was observed during the early post-dose period. Follow-up continued through 24 June 2026 (35 days post-dose), no gout flare recurred; however, biliary symptoms and a febrile episode with suspected catheter-related bloodstream infection occurred, with uncertain relationships to Firsekibart.

**Conclusion:**

In this maintenance hemodialysis patient, gout-related pain improved before the first post-dose dialysis session and did not rebound after dialysis. However, this single case, without pre- or post-dialysis Firsekibart concentration measurements, cannot establish causality or determine the effect of hemodialysis on drug exposure.

## 1 Introduction

Firsekibart is a fully human IgG4 monoclonal antibody targeting interleukin-1β (IL-1β), a central upstream mediator in the monosodium urate crystal-induced inflammatory cascade, thereby providing rapid control of inflammation and pain during an acute gout flare (Kim, 2022; Xue et al., 2025a; Xue et al., 2025b). Existing clinical studies have suggested that Firsekibart can rapidly relieve pain during acute gout flares and reduce the risk of subsequent recurrence. A randomized phase III trial further demonstrated that Firsekibart provides rapid pain relief and favorable short-term tolerability in patients with acute gout who are not candidates for standard therapy (Xue et al., 2025a; Xue et al., 2025b).

However, a significant evidence gap remains in literature. Patients receiving maintenance hemodialysis have been underrepresented in clinical studies evaluating the efficacy of Firsekibart. The supplemental materials of the pivotal phase III trial explicitly excluded patients receiving dialysis, and the product label does not provide dedicated pharmacokinetic data, optimal timing of drug administration, or efficacy and safety information in patients undergoing hemodialysis (Changchun Genescience Pharmaceuticals Co. Ltd., 2025). In patients with recent gastrointestinal bleeding, infection risk, severe anemia, and cardiovascular disease, the use of nonsteroidal anti-inflammatory drugs (NSAIDs), colchicine, and glucocorticoids may be limited or require careful consideration. In such settings, IL-1β-targeted therapies may be considered as a potential alternative treatment option, although evidence supporting their use in patients receiving hemodialysis remains limited (Richette et al., 2016; FitzGerald et al., 2020; Li et al., 2025).

Here, we report the clinical course of Firsekibart treatment for acute gouty arthritis in a patient receiving maintenance hemodialysis with a recent history of massive gastrointestinal bleeding, ongoing treatment for pulmonary infection, and high cardiovascular risk. The main observations were as follows: Firsekibart was administered between two hemodialysis sessions; pain improvement was documented before the first post-dose hemodialysis session; and no pain rebound was observed after the subsequent hemodialysis session. This report provides a descriptive observation of Firsekibart use and the timing of its administration relative to subsequent hemodialysis; however, it does not establish the effect of hemodialysis on Firsekibart exposure, pharmacokinetics, or pharmacodynamicactivity.

## 2 Case presentation

### 2.1 Baseline characteristics and comorbidities

The patient was a 51-year-old man with a history of coronary artery disease, hypertension, impaired glucose tolerance, hypertriglyceridemia, fatty liver disease, gallstones, thyroid nodules, chronic kidney disease stage 5, and gout. In February 2023, he underwent off-pump coronary artery bypass grafting for exertional chest tightness and chest pain, followed by long-term oral clopidogrel therapy.

The patient’s kidney disease history dated back to December 2022. He subsequently experienced multiple hospitalizations for persistently elevated serum creatinine levels, bilateral lower-limb edema, and foamy urine. On 1 May 2026, he was admitted to the intensive care unit (ICU) for massive gastrointestinal bleeding and underwent endoscopic electrocoagulation for hemostasis, followed by massive transfusion, continuous renal replacement therapy, anti-infective treatment, tracheal intubation, fasting, continuous gastrointestinal decompression, intensive acid suppression, somatostatin administration, and nutritional support.

The main diagnoses included massive gastrointestinal bleeding complicated by hemorrhagic shock; duodenal bulb ulcer status post embolization of the gastroduodenal artery, left gastric artery, and superior mesenteric artery; stage 5 chronic kidney disease; severe anemia; coronary atherosclerotic heart disease with unstable angina status post coronary artery bypass grafting; hyperuricemia with gouty arthritis; and pulmonary infection. After clinical stabilization, he was transferred to the Department of Nephrology, where maintenance hemodialysis and further treatment were continued.

### 2.2 History of gout

In 2018, the patient first developed erythema, swelling, and pain in the left first metatarsophalangeal joint. His serum uric acid level exceeded 600 μmol/L, and his symptoms resolved after symptomatic treatment. Thereafter, episodes of joint pain recurred more than 10 times per year and were relieved by self-administration of glucocorticoids and analgesics during acute flares. Beginning in January 2023, the patient experienced recurrent episodes of erythema, swelling, and pain involving both ankles, the right plantar arch, and the metatarsophalangeal joints, with symptom relief after self-administration of etoricoxib and colchicine. On 9 October 2025, he was hospitalized again because of erythema, swelling, and pain in the left elbow. Ultrasonography showed left elbow joint effusion and surrounding soft-tissue swelling. MRI showed a heterogeneous mass-like lesion in the posterior elbow soft tissues and a small joint effusion; the radiology report considered olecranon bursitis with possible infection and included a soft-tissue gouty tophus in the differential diagnosis (Figures 1a,b). On 22 October 2025, the patient underwent surgical excision of the posterior elbow soft-tissue lesion. No histopathology report for the excised tissue was available. After discharge in January 2026 and through the last follow-up on 24 June 2026, he continued regular maintenance hemodialysis but received no specific urate-lowering therapy. During the current hospitalization, sodium bicarbonate 1 g was administered orally three times daily from May 14 to May 29; this was not considered specific urate-lowering therapy. Before the current hospitalization, gout flares had occurred approximately once per month.

**FIGURE 1:**
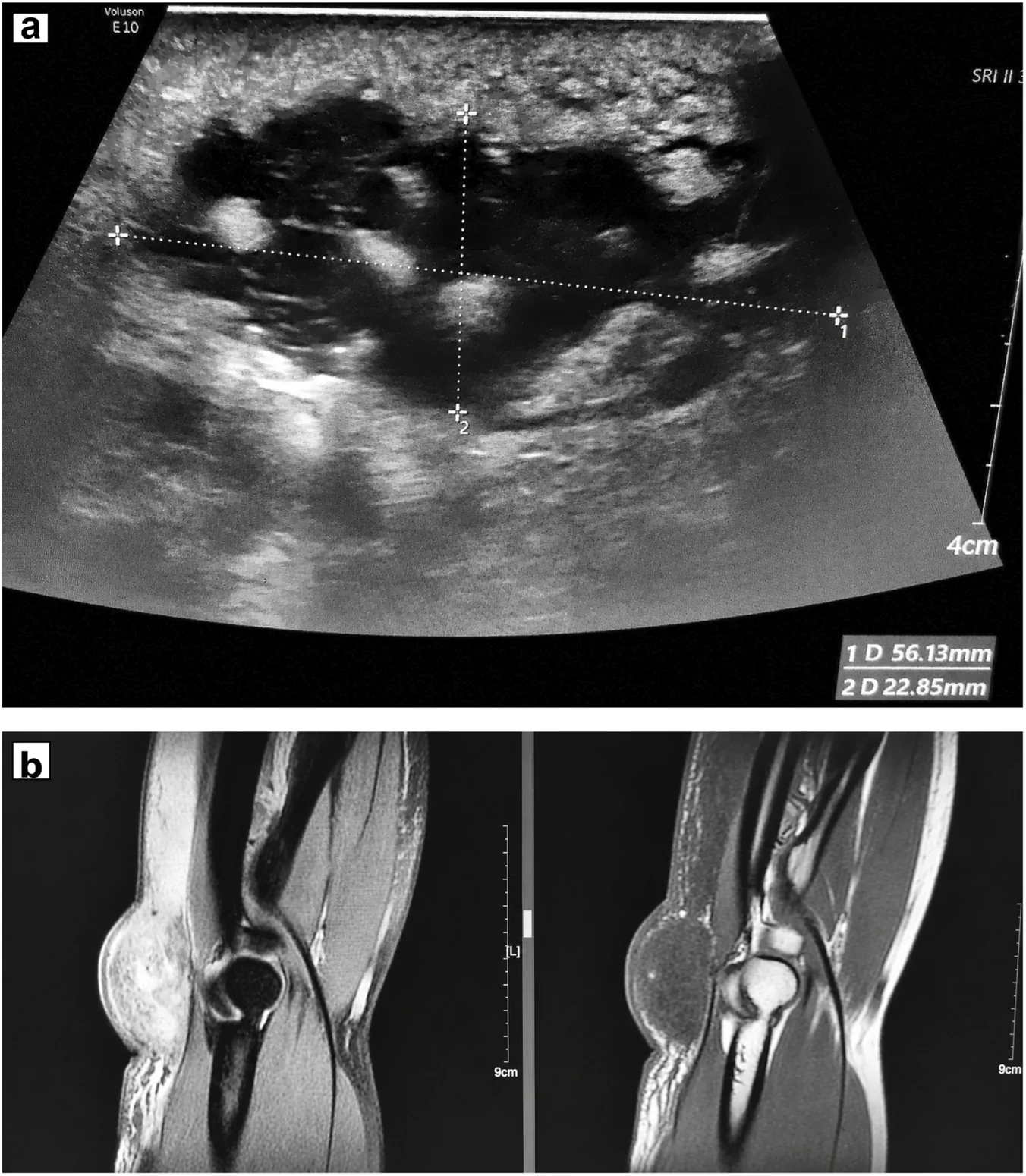
Imaging findings of the left elbow. **(a)** Superficial ultrasonography showed thickening of the subcutaneous soft tissues and an irregular fluid-containing area measuring approximately 56 × 23 mm with internal echogenic strands; the radiology report described left elbow joint effusion and soft-tissue swelling. **(b)** Magnetic resonance imaging of the left elbow showed a heterogeneous mass-like lesion in the posterior elbow soft tissues and a small joint effusion. The radiology report considered olecranon bursitis with possible infection and included a soft-tissue gouty tophus in the differential diagnosis. No histopathological confirmation was available.

### 2.3 Current acute gout flare and treatment

During the current hospitalization, beginning on 13 May 2026, the patient developed persistent erythema, swelling, and pain involving both knees, ankle joints, dorsum of the feet, and toe joints, accompanied by limited mobility and marked tenderness. His serum uric acid was 506 μmol/L. Considering his history of recurrent gout flares, prior imaging findings consistent with gout-related elbow involvement and a history of tophus removal, elevated serum uric acid, and the typical current manifestations of polyarticular erythema, swelling, warmth, and pain, an acute gout flare was clinically diagnosed.

All pre-Firsekibart anti-inflammatory and analgesic treatments were administered as temporary rescue therapy: tramadol sustained-release tablet 0.1 g orally on May 13; dexamethasone 5 mg intravenously on May 14; colchicine 0.5 mg plus etoricoxib 60 mg orally on May 18; tramadol 0.1 g intramuscularly on May 19; and, on May 20, topical flurbiprofen gel plasters at 16:11 and 17:33 and tramadol 0.1 g intramuscularly at 17:09. Pain relief remained insufficient.

No affected joint was aspirated; therefore, neither monosodium urate crystal analysis nor synovial fluid culture was performed. No additional imaging of the currently affected joints, including ultrasonography, magnetic resonance imaging, or dual-energy computed tomography, was obtained, and no blood culture was performed in the period around May 20. Because of the concurrent pulmonary infection, septic arthritis could not be definitively excluded. Similarly, other crystal arthropathies could not be excluded in the absence of synovial fluid crystal analysis. The diagnosis of an acute gout flare was therefore made clinically, based on the patient’s recurrent history of gout flares, prior elbow imaging showing olecranon bursitis with possible tophaceous involvement, hyperuricemia, and the current polyarticular presentation.

At the time of Firsekibart administration, the pulmonary infection was still being treated with ceftazidime (May 13–June 4) and levofloxacin (May 13–29), and sputum culture had grown multidrug-resistant *Pseudomonas aeruginosa*. On May 20, the patient had no obvious cough or sputum production, and his recorded temperature before administration ranged from 36.1 °C to 36.9 °C. However, there was no documented microbiological confirmation of eradication at the time of Firsekibart administration.

Given the patient’s stage 5 chronic kidney disease necessitating maintenance hemodialysis, recent severe gastrointestinal bleeding, severe anemia, pulmonary infection, and high cardiovascular-risk status, NSAIDs, colchicine, and glucocorticoids were considered to have substantial limitations. The decision to administer firsekibart therefore reflected a case-specific risk-benefit assessment in the setting of persistent, inadequately controlled gout symptoms and limited conventional treatment options, while recognizing that an active or incompletely controlled infection could not be excluded. Because of persistent pain despite these treatment limitations, a single 200-mg dose of Firsekibart was administered subcutaneously at 17:58 on 20 May 2026, while antimicrobial therapy continued. No additional dose of these anti-inflammatory or analgesic agents were administered after Firsekibart. However, because tramadol and topical flurbiprofen had been administered within the preceding hour, their effects may have overlapped with the early post-dose period, and their potential contribution to the subsequent improvement in pain documented 6–7 h later cannot be excluded.

## 3 Temporal relationship between dialysis and drug administration and clinical course

A notable feature of this case was that Firsekibart was administered between two hemodialysis sessions, with pain relief first documented before the first post-dose hemodialysis session. The detailed temporal relationship is presented in Table 1.

**TABLE 1:** Treatment, hemodialysis, and follow-up timeline.

Time	Event	Dialysis/treatment information
18 May 2026	Last hemodialysis session before drug administration	4-h hemodialysis; net ultrafiltration 1,500 mL; MF16 dialyzer
20 May 2026, 16:11–17:33	Rescue treatment immediately before Firsekibart	Topical flurbiprofen gel plasters at 16:11 and 17:33; tramadol 0.1 g intramuscularly at 17:09
20 May 2026, 17:58	Firsekibart administration	200 mg (single subcutaneous injection)
6–7 h after drug administration	Pain began to improve	No post-dose hemodialysis had yet been performed
21 May 2026	First post-administration hemodialysis session	4-h hemodialysis; net ultrafiltration 1700 mL; B18HF dialyzer
Days 4–10 after drug administration	Sustained clinical improvement	VAS decreased from 2 to 0
28 May 2026 (day 8)	Epigastric/subxiphoid pain and diarrhea	CT: Cholelithiasis with possible cholecystitis; ceftazidime continued
June 3–9, 2026 (days 14–20)	Fever, chills, and clinically suspected catheter-related bloodstream infection	Blood culture: *Staphylococcus* lugdunensis; empiric ertapenem; catheter removed June 4 and reinserted June 5; no further fever; discharged June 9
11 June 2026 (day 22)	Recurrent upper abdominal pain without fever	MRI: Cholelithiasis with cholecystitis and a folded gallbladder; symptomatic treatment
24 June 2026 (day 35)	Last documented clinical follow-up	No recurrent acute gout flare documented

Abbreviations: VAS, visual analogue scale; h, hours.

Before administration, the patient exhibited marked erythema, swelling, and pain in both knees and feet, with a VAS pain score of 10. Pain improvement was first documented approximately 6–7 h after Firsekibart administration, although the potential contribution of tramadol and topical flurbiprofen given shortly before Firsekibart cannot be excluded. The VAS score was recorded as 8 on day 1, 2 on day 4, 1 on day 7, and 0 on day 10 after administration, with gradual recovery from marked mobility limitation to ambulation. Following the first post-dose hemodialysis session on May 21, no exacerbation or rebound of pain was observed on the day of dialysis or the following day. Details are shown in Table 2.

**TABLE 2:** Symptom changes after the current acute gout flare.

Time point	VAS pain score	Joint manifestations
Prior to drug administration on May 20	10	Marked erythema, swelling, pain, and tenderness involving both knees and feet
6–7 h after drug administration	NA	Pain began to improve
Day 1 after drug administration	8	Marked improvement in pain observed, but swelling and impaired mobility persisted
Day 4 after drug administration	2	Pain had largely subsided, with persistent swelling and limited mobility
Day 7 after drug administration	1	Mild swelling; able to move in bed
Day 10 after drug administration	0	No obvious swelling; able to ambulate

Abbreviations: VAS, visual analogue scale; NA, not available.

Tramadol and topical flurbiprofen were administered within the hour before Firsekibart and may have contributed to the pain improvement first documented 6–7 h later.

The above laboratory results showed that WBC decreased from 8.12 × 10^9^/L before administration to 4.56 × 10^9^/L on day 1 and 4.30 × 10^9^/L on day 4 after administration. The absolute neutrophil count decreased from 7.20 × 10^9^/L to 3.33 × 10^9^/L on day 1 and 3.11 × 10^9^/L on day 4. PCT decreased from 6.66 ng/mL to 1.48 ng/mL on day 4 and 0.51 ng/mL on day 7. Serum uric acid gradually decreased from 506 μmol/L to 429 μmol/L on day 7 and then 329 μmol/L in early June. CRP decreased from 105.98 mg/L before administration to 10.48 mg/L in early June. The patient’s body temperature ranged from 36.1 °C–36.9 °C before administration. Following administration on May 20, it transiently increased to 38.4 °C before subsequently decreasing. By May 21, body temperature ranged from 36.2 °C–36.6 °C. No persistent fever was observed on days 3–4 after administration. No acute hypersensitivity reaction, including rash, pruritus, chest tightness, dyspnea, or chills, was observed during the early post-dose period. Details are shown in Table 3.

**TABLE 3:** Major laboratory changes before and after drug administration.

Parameter	Before administration on May 20	Day 1 after administration	Day 4 after administration	Day 7 after administration	Early June
WBC (×10^9^/L)	8.12	4.56	4.30	4.58	5.04
NEUT# (×10^9^/L)	7.20	3.33	3.11	3.33	NA
CRP (mg/L)	105.98	NA	NA	NA	10.48
PCT (ng/mL)	6.66	NA	1.48	0.51	1.99
SUA (μmol/L)	506	NA	NA	429	329
ALT (U/L)	8	NA	NA	20	16

Abbreviations: ALT, alanine aminotransferase; CRP, C-reactive protein; NA, not available; NEUT#, absolute neutrophil count; PCT, procalcitonin; SUA, serum uric acid; WBC, white blood cell count.

Hemodialysis was continued as scheduled after Firsekibart administration, and the patient continued regular maintenance hemodialysis. Subsequent clinical records showed no exacerbation of joint pain or rebound in the VAS score after hemodialysis. The documented joint pain and mobility limitation gradually improved, with the VAS score eventually reaching 0. No further acute gout flares were documented through 24 June 2026 (day 35 after administration).

## 4 Follow-up of other clinical manifestations

On May 28 (day 8 after administration), the patient developed persistent epigastric/subxiphoid pain and diarrhea following hemodialysis; WBC was 4.85 × 10^9^/L, neutrophil percentage was 75.7%, and PCT was 0.35 ng/mL. Abdominal CT showed cholelithiasis with possible cholecystitis, and ceftazidime was continued. On June 3 (day 14 after administration), the patient developed high fever and chills; WBC was 5.04 × 10^9^/L, neutrophil percentage was 90.8%, and PCT was 1.99 ng/mL blood culture was positive, initially showing Gram-positive cocci and subsequently identified as *Staphylococcus* lugdunensis. Antimicrobial therapy was empirically changed to ertapenem, and a catheter-related bloodstream infection was clinically suspected. The temporary internal jugular dialysis catheter was removed on June 4, and a new internal jugular dialysis catheter was inserted on June 5; no further fever was documented thereafter. The patient was discharged on June 9 without abdominal pain or fever. On June 11 (day 22 after administration), upper abdominal pain recurred without fever, jaundice, or chills. MRI showed cholelithiasis with cholecystitis and a folded gallbladder, and symptomatic treatment was provided. No recurrent acute gout flare was documented through the last follow-up on June 24.

## 5 Patient perspective

The patient recalled that the pain during this acute gout flare was almost unbearable; even a light breeze over the affected joints or the slightest movement caused severe pain. He felt that the conventional analgesic treatments administered before firsekibart provided little noticeable relief. Over the several days following firsekibart administration, he noticed a marked reduction in pain and was soon able to get out of bed and walk again. He was very satisfied with the improvement and spoke positively about the treatment based on his personal experience.

## 6 Discussion

Firsekibart has a scientific rationale for use in the treatment of acute gouty arthritis, supported by available clinical evidence. Previous clinical studies have reported anti-inflammatory and analgesic effects during acute gout flares and a lower risk of subsequent flares (Xue et al., 2025a). A randomized phase III study comparing it with compound betamethasone reported rapid pain relief and a lower risk of recurrent flares in patients with acute gout who were unsuitable for conventional anti-inflammatory therapy (Xue et al., 2025b). These studies provide clinical evidence supporting the efficacy of Firsekibart for the treatment of acute gout in the populations studied. Additional evidence is available from a post-hoc analysis presented at ACR Convergence 2025, which included 42 patients with eGFR <60 mL/min/1.73 m^2^ and reported similar 72-h pain reduction with Firsekibart compared with compound betamethasone, fewer recurrent flares through 24 weeks, and no serious adverse events among patients who received Firsekibart (Xue et al., 2025a).

However, these patients in this analysis had non-dialysis-dependent CKD, so these findings cannot be directly extrapolated to patients receiving maintenance hemodialysis. Evidence regarding the use of Firsekibart in the maintenance hemodialysis population remains limited. The supplemental materials of the pivotal phase III trial listed renal dialysis as an exclusion criterion (Xue et al., 2025c). The product label also reports only limited experience in patients with mild-to-moderate renal impairment and does not provide dedicated pharmacokinetic data, data on the effect of hemodialysis on Firsekibart exposure, or safety data for patients receiving maintenance hemodialysis (Changchun Genescience Pharmaceuticals Co. Ltd., 2025). Therefore, the timing of clinical response to Firsekibart, the persistence of its clinical effect across hemodialysis sessions, and its safety in patients with high risk of infection remain to be determined.

Because direct evidence on Firsekibart in patients receiving maintenance hemodialysis is limited, experience with other IL-1 pathway agents in patients with advanced chronic kidney disease or receiving dialysis may provide relevant clinical context when considering its potential use. A multicenter retrospective study by Loustau et al. included patients with stage 4–5 chronic kidney disease or kidney transplantation and reported that the IL-1 receptor antagonist anakinra may be a therapeutic option for patients in whom conventional anti-inflammatory therapies are limited, with clinical responses and safety findings that were considered acceptable (L et al., 2018). The case series and scoping review by Jeria-Navarro et al. further summarized the available clinical evidence on anakinra in complex clinical settings, including refractory gout, concurrent infection, maintenance hemodialysis, kidney transplantation, and polyarticular involvement. The findings suggest that IL-1 pathway inhibition may be a therapeutic consideration in selected high-risk patients with gout flares (Jeria-Navarro et al., 2023). Nevertheless, anakinra and firsekibart differ in molecular structure, mechanism of action, half-life, and administration pattern; therefore, the efficacy and safety findings observed with of anakinra cannot be directly extrapolated to firsekibart. These data nevertheless suggest that targeting the IL-1 pathway may be a clinically relevent therapeutic approach in selected patients with advanced CKD or receiving dialysis, particularly when conventional anti-inflammatory therapies are limited. As a long-acting anti-IL-1β monoclonal antibody, firsekibart requires further evaluation in the hemodialysis population through additional real-world clinical experience, pharmacokinetic studies, and prospective research.

More recently, Wei et al. reported the use of firsekibart in a patient receiving peritoneal dialysis combined with intermittent hemodialysis, with rapid improvement in symptoms and no recurrent acute gout flare documented during follow-up (Wei et al., 2026). Although this setting differs from maintenance hemodialysis, the report provides relevant clinical experience with firsekibart in a dialysis patient. Canakinumab, like firsekibart, is a monoclonal antibody targeting IL-1β. In two randomized trials in patients with limited treatment options, canakinumab provided greater 72-h pain relief than triamcinolone acetonide and reduced the risk of new flares; infections, neutropenia, and thrombocytopenia were reported more frequently with canakinumab (Schlesinger et al., 2012). These findings provide additional clinical support for IL-1β as a therapeutic target, but the efficacy and safety findings cannot be directly extrapolated to firsekibart or to patients receiving maintenance hemodialysis.

Accordingly, the novelty of the present report lies primarily in the clinical experience with firsekibart in a patient receiving maintenance hemodialysis and the documented timing of firsekibart administration relative to the subsequent hemodialysis session, rather than in the use of firsekibart in a dialysis patient *per se*.

This observation that pain improvement was documented before the first post-dose hemodialysis session and that no pain rebound was observed after that session is pharmacokinetically plausible based on the known properties of monoclonal antibodies and conventional hemodialysis membranes. The solute clearance capacity of hemodialysis is influenced by dialyzer membrane characteristics, including membrane material, surface area, flux, ultrafiltration coefficient, and sieving properties. The MF16 used in this case is a medium-flux dialyzer, whereas the B18HF is a high-flux dialyzer; both are primarily designed to clear small molecules and selected middle-molecular-weight uremic toxins. Firsekibart is a macromolecular protein drug with a molecular weight of approximately 150 kDa, making substantial removal by conventional hemodialysis membranes theoretically unlikely. In general, monoclonal antibodies are eliminated primarily through the reticuloendothelial system, target-mediated clearance, and intracellular proteolysis rather than through glomerular filtration and renal excretion. Conventional hemodialysis or peritoneal dialysis is therefore not expected to result in substantial clearance of macromolecular monoclonal antibodies (Mansour et al., 2023). Therefore, the pharmacodynamic effect of a single subcutaneous injection may theoretically persist across subsequent dialysis sessions. Together with the absence of pain rebound after hemodialysis on the day following administration and the continued improvement in inflammatory markers and clinical symptoms, these observations are consistent with, but do not establish, the possibility that the dialysis session did not significantly attenuate the short-term clinical effect of firsekibart.

At the same time, the potential relationship between hemodialysis and the clinical response of firsekibart should not be considered solely from a drug-clearance perspective. Patients receiving maintenance hemodialysis may experience a chronic low-grade inflammatory state related to multiple factors, including the accumulation of uremic toxins, exposure to dialysis membranes, oxidative stress, malnutrition, and infection risk (Wang and Gao, 2022). Regular hemodialysis can remove uric acid, uremic toxins, and certain small-molecular inflammatory mediators, thereby modifying the uremia-related internal milieu. Firsekibart, in contrast, exerts its therapeutic effect through targeted blockade of the IL-1β-mediated inflammatory pathway. Thus, the two interventions act through different mechanisms and may theoretically have complementary effects, with hemodialysis modifying the systemic milieu while firsekibart targets a specific inflammatory pathway. In this case, while the patient continued regular hemodialysis, joint pain and mobility gradually improved after firsekibart administration, with concurrent improvement in inflammatory markers; no post-dialysis pain rebound was observed. These findings are compatible with, but do not establish, the possibility that hemodialysis and firsekibart may exert complementary effects through their distinct mechanisms. However, because firsekibart blood concentrations before and after dialysis were not measured and inflammatory mediators such as IL-1β were not continuously assessed, the present case cannot determine whether hemodialysis affected firsekibart exposure or pharmacodynamic activity. Therefore, in this case, no apparent attenuation of the short-term clinical effect of firsekibart was observed after subsequent hemodialysis, although the effect of dialysis on firsekibart exposure remains unknown.

The laboratory findings should be interpreted with caution. Although WBC count, absolute neutrophil count, PCT, subsequent CRP levels, and serum uric acid generally decreased after firsekibart administration, these changes occurred in the context of concurrent anti-infective therapy, blood transfusion, nutritional support, gastroprotective treatment, and regular hemodialysis, which introduced substantial potential confounding. PCT primarily reflects the degree of infection or systemic inflammatory burden and cannot substitute for CRP or clinical assessment of arthritis activity. Moreover, continuous monitoring of CRP after firsekibart administration was not performed in this case. Therefore, the observed laboratory changes should be regarded only as supportive findings consistent with clinical improvement and should not be interpreted as evidence of firsekibart efficacy in isolation.

In terms of safety, no definite acute hypersensitivity reactions, including rash, pruritus, chest tightness, dyspnea, or chills, were observed during the early post-administration period. No persistent high-grade fever was observed on days 3–4 after administration.

Later clinical events require separate consideration. Abdominal symptoms on day 8 were accompanied by imaging evidence of cholelithiasis with possible cholecystitis. The febrile episode on day 14 occurred in the setting of a positive blood culture for *Staphylococcus lugdunensis* and was clinically managed as a suspected catheter-related bloodstream infection; no further fever occurred after antimicrobial adjustment and catheter replacement. Upper abdominal pain recurred on day 22, with MRI evidence of cholelithiasis and cholecystitis. Given the pre-existing pulmonary infection, structural biliary disease, temporary dialysis catheter, prolonged hospitalization, maintenance hemodialysis, and multiple invasive procedures, a causal contribution of firsekibart to these events can neither be established nor excluded. Overall, this case only indicates that no definite immediate hypersensitivity reaction was observed during the early post-administration period and that no pain rebound was observed after subsequent hemodialysis. It cannot establish the safety of firsekibart in patients with complex comorbidities receiving maintenance hemodialysis.

Several limitations should be considered when interpreting this case. First, as a single case report, the level of evidence is limited, and the findings cannot establish causality or support generalizable conclusions. Second, synovial fluid was not obtained from the currently affected joints for monosodium urate crystal analysis, and ultrasonography or dual-energy computed tomography of the currently involved joints was not performed. Third, serial measurements of CRP, IL-1β, and other inflammatory mediators after firsekibart administration were not available, limiting direct assessment of changes in the IL-1β-mediated inflammatory pathway. Fourth, Firsekibart concentrations before and after hemodialysis were not measured; therefore the effect of hemodialysis on firsekibart exposure could not be evaluated pharmacokinetically. Fifth, the patient received concurrent anti-infective therapy, blood transfusions, nutritional support, regular hemodialysis, and multiple symptomatic treatments, introducing substantial potential confounding. Finally, follow-up was limited to 35 days after firsekibart administration; therefore, delayed adverse events, longer-term recurrence, the need for repeat dosing, and long-term safety could not be evaluated.

## 7 Conclusion

This case describes firsekibart use in a patient receiving maintenance hemodialysis and documents the timing of firsekibart administration relative to the subsequent hemodialysis session. Pain continued to improve, with no rebound observed after that session. However, because this is a single case, pre- and post-dialysis firsekibart concentration were not measured, and analgesic and anti-inflammatory treatments were administered shortly before firsekibart in the setting of concomitant infection, neither causality nor the effect of hemodialysis on firsekibart exposure or pharmacodynamic activity can be determined. Additional real-world clinical experience, pharmacokinetic studies with measurement of blood firsekibart concentrations before and after hemodialysis, and prospective studies are needed to clarify the pharmacokinetic characteristics and safety of firsekibart in patients receiving maintenance hemodialysis.

## Data Availability

The original contributions presented in the study are included in the article/supplementary material, further inquiries can be directed to the corresponding author.
